# Association of Disability With Mortality From Opioid Overdose Among US Medicare Adults

**DOI:** 10.1001/jamanetworkopen.2019.15638

**Published:** 2019-11-15

**Authors:** Yong-Fang Kuo, Mukaila A. Raji, James S. Goodwin

**Affiliations:** 1 Sealy Center on Aging, Department of Internal Medicine, The University of Texas Medical Branch at Galveston, Galveston; 2 Department of Preventive Medicine and Population Health, The University of Texas Medical Branch at Galveston, Galveston; 3 Institute for Translational Science, The University of Texas Medical Branch at Galveston, Galveston

## Abstract

**Importance:**

Patients qualifying for Medicare disability have the highest rates of opioid use compared with older Medicare beneficiaries and commercial insurance beneficiaries. Research on opioid overdose deaths in this population can help identify appropriate interventions.

**Objective:**

To assess the rate of opioid overdose death and to identify its associated risk factors.

**Design, Setting, and Participants:**

This cohort study included a 20% national sample of Medicare enrollees aged 21 to 64 years whose initial Medicare entitlement was based on disability and who resided in 50 US states and Washington, DC, in 2012 to 2016. Data analyses were performed from March 15, 2019, through September 23, 2019.

**Exposures:**

Fifty-five chronic or potentially disabling conditions were selected from the Centers for Medicare & Medicaid Services Chronic Disease Data Warehouse.

**Main Outcomes and Measures:**

Opioid overdose death rate estimated from Medicare National Death Index linkage data.

**Results:**

Among 1 766 790 Medicare enrollees younger than 65 years who qualified for Medicare because of disability, the mean (SD) age was 52.2 (10.2) years, and 866 914 (49.1%) were women. These enrollees represent 14.9% (95% CI, 14.9%-15.0%) of the entire Medicare population and accounted for 80.8% (95% CI, 78.9%-82.7%) of opioid overdose deaths among all Medicare enrollees. Opioid overdose mortality in this population increased from 57.4 per 100 000 (95% CI, 53.9-61.0 per 100 000) in 2012 to 77.6 per 100 000 (95% CI, 73.5-81.8 per 100 000) in 2016. Results from the stepwise logistic regression model revealed that 3 categories of conditions are associated with opioid overdose death: substance abuse, psychiatric diseases, and chronic pain. Among the 11.1% (95% CI, 11.0%-11.2%) of adults with disability who had all 3 conditions, the rate of opioid overdose death was 363.7 per 100 000 (95% CI, 326.7-402.6 per 100 000), which is 23.4 times higher than the rate for individuals with none of the conditions (15.5 per 100 000; 95% CI, 11.6-20.1 per 100 000).

**Conclusions and Relevance:**

This study identifies differences in opioid overdose mortality among subgroups of Medicare enrollees younger than 65 years who qualify for Medicare because of disability. Understanding the heterogeneity of medical and psychiatric conditions associated with opioid use and misuse is key to developing specific, data-driven interventions targeted to each subgroup of high-risk populations.

## Key Points

**Question:**

What is the rate of opioid overdose deaths among Medicare enrollees younger than 65 years who qualified for Medicare because of disability?

**Findings:**

In this cohort study, the 1 766 790 adult Medicare enrollees who qualified for disability—representing 14.9% of the Medicare population—accounted for 80.8% of all opioid overdose deaths among all Medicare enrollees. Among the 11.1% of the enrollees with disability who had 3 co-occurring conditions of substance abuse, psychiatric diseases, and chronic pain syndrome, the opioid overdose death rate was 23.4 times higher than that for enrollees without any of the conditions.

**Meaning:**

Understanding the heterogeneity of medical and psychiatric conditions associated with opioid use and misuse is key to developing specific, data-driven interventions targeted for each subgroup of high-risk populations.

## Teaser

This cohort study examines the rate of opioid overdose deaths among Medicare recipients younger than 65 years who qualified for Medicare because of disability.

## Introduction

Medicare beneficiaries who qualify for Medicare because of disability constitute a growing population of patients hospitalized for opioid or heroin overdose^1,2^ and account for 25% of deaths from prescription opioid overdose annually.^3^ The Centers for Disease Control and Prevention (CDC) regularly generates reports of opioid overdose deaths by demographic variables and states, but studies on policy-actionable risk factors (eg, clusters of medical and psychiatric conditions or types of disabling conditions) for overdose mortality are lacking in this population.

## Methods

The University of Texas Medical Branch institutional review board approved this study and waived any informed consent requirement because the research used deidentified data. This study follows the Strengthening the Reporting of Observational Studies in Epidemiology (STROBE) reporting guideline.^4^

Our initial cohort was a 20% national sample of all Medicare enrollees aged 21 to 100 years residing in 50 US states and Washington, DC, in 2012 to 2016. Centers for Medicare & Medicaid Services (CMS) selects a random sample of 20% Medicare beneficiaries according to the last 2 digits of their health insurance claim number. Studies based on these data should provide generalizable estimates for the Medicare population.^5^ We used Medicare summary files linked to the National Death Index. From the National Death Index, we identified drug overdose deaths according to *International Statistical Classification of Diseases and Related Health Problems*,* Tenth Revision*, codes for cause of death: X40-44, X60-64, X85, or Y10-Y14. Opioid overdose deaths were identified by codes T40.0 (opium), T40.1 (heroin), T40.2 (natural or semisynthetic opioids), T40.3 (methadone), T40.4 (synthetic opioids other than methadone), or T40.6 (other and unspecified narcotics) from the underlying conditions.

Patient characteristics included age, sex, race/ethnicity, years under Medicare disability entitlement, health maintenance organization enrollment, and Medicare Part D enrollment for at least 1 month in a year. Race/ethnicity is based on Social Security Administration data, with a surname algorithm enhancing the identification of Hispanic and Asian origin.^6^ Medicaid enrollment was used to represent low income. Education and household income were obtained from the 2011 to 2015 American Community Survey by zip code linkage and were divided into quartiles. Residential area was classified using rural-urban continuum codes.^7^

The CMS Chronic Conditions Data Warehouse listed 27 chronic conditions and 36 potentially disabling conditions as of March 2019. Chronic Conditions Data Warehouse is a research database designed to improve the quality of care and reduce costs and utilization. The specific definition and reference for each condition are available at the Chronic Conditions Data Warehouse website.^8^ We combined these 2 sets of conditions, removed the duplicate conditions (eg, depression and depressive disorder), and consolidated the cancer diagnoses (breast, lung, colorectal, endometrial, prostate cancer, or leukemia and lymphomas). This resulted in 55 chronic or potentially disabling conditions for our study (Table 1).

**Table 1.** Comparison of Demographic Characteristics and Medical Conditions Between All Medicare Enrollees Aged 21 to 64 Years Qualified for Disability in 2016 and Those Who Died of Opioid Overdose

Variables	Participants, No. (%)		*P* Value^a^
	Adult Medicare Recipients Qualified for Disability (N = 1 766 790)	Opioid Overdose Death (n = 1371)	
Demographic characteristics			
Age, y			
21-30	84 470 (4.8)	53 (3.9)	<.001
31-40	186 323 (10.6)	234 (17.1)	
41-50	328 539 (18.6)	328 (23.9)	
51-64	1 167 458 (66.1)	756 (55.1)	
Mean (SD)	52.2 (10.2)	49.7 (9.9)	
Sex			
Male	899 876 (50.9)	773 (56.4)	.004
Female	866 914 (49.1)	598 (43.6)	
Race/ethnicity			
White	1 170 832 (66.3)	1114 (81.3)	<.001
Black	350 744 (19.9)	127 (9.3)	
Hispanic	172 862 (9.8)	76 (5.5)	
Other	72 352 (4.1)	54 (3.9)	
Years under disability insurance			
≤4	524 930 (29.7)	366 (26.7)	<.001
5-8	509 017 (28.8)	494 (36.0)	
9-14	310 789 (17.8)	282 (20.6)	
≥15	422 954 (23.9)	229 (16.7)	
Mean (SD)	10.2 (8.4)	9.0 (6.4)	
Dual eligible			
Yes	866 544 (49.0)	796 (58.1)	<.001
No	900 246 (51.0)	575 (41.9)	
Zip code education^b^			
Quartile 1 (lowest)	577 915 (33.1)	362 (26.7)	<.001
Quartile 2	524 919 (30.1)	442 (32.6)	
Quartile 3	379 445 (21.7)	322 (23.7)	
Quartile 4 (highest)	263 807 (15.1)	230 (17.0)	
Zip code income^b^			
Quartile 1 (lowest)	612 138 (34.8)	432 (31.6)	.02
Quartile 2	488 192 (27.7)	355 (25.9)	
Quartile 3	391 224 (22.2)	323 (23.6)	
Quartile 4 (highest)	268 458 (15.3)	259 (18.9)	
Rural vs urban^b^			
Metropolitan area	1 403 542 (79.5)	1178 (85.9)	<.001
Urban	324 070 (18.4)	170 (12.4)	
Rural	37 899 (2.1)	23 (1.7)	
HMO enrollment			
Yes	509 657 (28.8)	338 (24.6)	.04
No	1 257 133 (71.2)	1033 (75.4)	
Medicare Part D enrollment			
Yes	1 412 948 (80.0)	1205 (87.9)	<.001
No	353 842 (20.0)	166 (12.1)	
Medical condition diagnoses^c^			
Psychiatric diseases			
Depression	264 809 (30.2)	436 (56.8)	<.001
Anxiety	279 297 (30.9)	487 (63.4)	<.001
Bipolar disorder	122 830 (14.0)	273 (35.6)	<.001
Schizophrenia and other psychotic disorders	100 784 (11.5)	144 (18.8)	<.001
Attention-deficit/hyperactivity disorder	39 812 (4.6)	92 (12.0)	<.001
Autism	12 218 (1.4)	NA^d^	
Personality disorders	35 766 (4.1)	75 (9.8)	<.001
Posttraumatic stress disorder	44 306 (5.1)	94 (12.2)	<.001
Development disorder			
Intellectual disabilities	54 008 (6.2)	NA^d^	
Learning disability	5112 (0.6)	NA^d^	
Other developmental delays	6322 (0.7)	NA^d^	
Cystic fibrosis and other metabolic developmental disorders	9077 (1.0)	13 (1.7)	>.99
Substance abuse			
Tobacco use disorder	207 378 (23.7)	437 (56.9)	<.001
Alcohol use disorder	58 582 (6.7)	186 (24.2)	<.001
Drug use disorder	105 625 (12.1)	432 (56.3)	<.001
Any OUD^e^	63 307 (7.2)	389 (50.7)	<.001
OUD diagnosis or procedure	54 441 (6.2)	351 (45.7)	<.001
OUD emergency department or hospitalization^e^	30 605 (3.5)	315 (41.0)	<.001
Use of medication-assisted treatment^e^	11 624 (1.3)	82 (10.7)	<.001
Disability-related conditions			
Cerebral palsy	17 024 (1.9)	NA^d^	
Epilepsy	69 847 (8.0)	86 (11.2)	.07
Deafness and hearing impairment	25 825 (3.0)	11 (1.4)	.59
Mobility impairment	38 764 (4.4)	38 (5.0)	>.99
Multiple sclerosis and transverse myelitis	17 747 (2.0)	NA^d^	
Muscular dystrophy	2243 (0.3)	NA^d^	
Spina bifida and other congenital anomalies of the nervous system	5364 (0.6)	NA^d^	
Spinal cord injury	7068 (0.8)	12 (1.6)	.73
Blindness and visual impairment	7919 (0.9)	NA^d^	
Traumatic brain injury	8248 (0.9)	NA^d^	
Pain diagnosis			
Migraine and other chronic headache	65 303 (7.5)	98 (12.8)	<.001
Fibromyalgia, chronic pain, and fatigue	271 913 (31.1)	489 (63.7)	<.001
Chronic conditions			
Hypertension	368 959 (42.1)	281 (36.6)	.12
Heart failure	82 239 (9.4)	90 (11.7)	.85
Ischemic heart disease	145 398 (16.6)	150 (19.5)	.87
Acute myocardial infarction	5270 (0.6)	NA^d^	
Atrial fibrillation	18 905 (2.2)	11 (1.4)	>.99
Hyperlipidemia	276 283 (31.6)	136 (17.7)	<.001
Peripheral vascular disease	65 341 (7.5)	44 (5.7)	>.99
Stroke	22 659 (2.6)	27 (3.5)	>.99
Arthritis	250 922 (28.7)	297 (38.7)	<.001
Asthma	73 603 (8.4)	75 (9.8)	>.99
Cancer	34 758 (4.0)	16 (2.1)	.40
Benign prostatic hyperplasia	23 466 (2.7)	15 (2.0)	>.99
Chronic kidney disease	156 158 (17.8)	190 (24.7)	<.001
Chronic obstructive pulmonary disease	112 848 (12.9)	133 (17.3)	.02
Dementia^f^	34 062 (4.4)	28 (4.2)	>.99
Diabetes	231 423 (26.4)	186 (24.2)	>.99
Obesity	210 268 (24.0)	177 (23.1)	>.99
Anemia	156 457 (17.9)	143 (18.6)	>.99
Hypothyroidism	100 638 (11.5)	57 (7.4)	.03
Cataract	54 849 (6.3)	16 (2.1)	<.001
Glaucoma	25 932 (3.0)	NA^d^	
Osteoporosis	22 700 (2.6)	13 (1.7)	>.99
Hip or pelvic fracture	1887 (0.2)	NA^d^	
Pressure and chronic ulcers	39 560 (4.5)	61 (7.9)	<.001
Liver diseases	55 939 (6.4)	105 (13.7)	<.001
Hepatitis	33 977 (3.9)	133 (17.3)	<.001
HIV	15 439 (1.8)	19 (2.5)	>.99

Abbreviations: HMO, health maintenance organization; NA, not applicable; OUD, opioid use disorder.

^a^ *P* value from goodness-of-fit test with Bonferroni correction for multiple comparisons. *P* values shown have been calculated by using the following formula: 1 – (1 – original *P* value)^57^. There are a total of 68 comparisons in this table.

^b^ Variables had missing data.

^c^ Data for medical conditions are shown only for 875 682 Medicare recipients who qualified for disability, had 2 years of continuous enrollment in Medicare Part A and B, and were not members of HMOs in 2015 and 2016; 768 died from opioid overdose.

^d^ Thirteen conditions associated with fewer than 11 deaths from opioid overdose were not included because of the CMS cell size suppression policy to protect the confidentiality of enrollees.

^e^ Not the condition in the Chronic Conditions Data Warehouse. Only for descriptive statistics.

^f^ Required 3 years of continuous enrollment in Medicare Part A and B without HMO coverage.

### Statistical Analysis

We calculated the number of deaths from opioid overdose among all Medicare recipients aged 21 to 100 years and the percentage of opioid overdose deaths by age and by reason for initial entitlement (age, disability, or end-stage renal disease) in 2016. Confidence intervals of these percentages were calculated according to multinomial distribution.^9^ Our main analyses focused on enrollees aged 21 to 64 years who initially qualified for Medicare because of disability. We first calculated the rate of opioid overdose deaths from 2012 to 216. Then we restricted the analyses to the 2016 data to compare their characteristics with those who died from opioid overdose, in bivariate analyses using goodness-of-fit test, and reported *P* values with Bonferroni correction for the multiple comparisons. For multivariable analyses, only those with Medicare Part A and B continuous enrollment and without health maintenance organization coverage in 2015 and 2016 were included and analyzed, by a stepwise logistic regression model, to identify conditions and characteristics associated with opioid overdose deaths. Thirteen conditions associated with fewer than 11 deaths from opioid overdose were not included because of the CMS cell size suppression policy to protect the confidentiality of enrollees. No obvious multicollinearity was found, with all correlations among characteristics and study conditions being less than 0.38. On the basis of the results of stepwise logistic regression, we grouped the conditions that were positively associated with opioid overdose death into 3 major common categories of conditions: substance abuse, psychiatric disease, and chronic pain. We analyzed the overlap across these 3 categories to show the proportion of enrollees and opioid overdose mortality for their combinations. The 95% CI of opioid overdose mortality was calculated assuming a Poisson distribution. In addition, we repeated analyses using the 2012 to 2015 data for 2 purposes: to validate our final model in the 2016 data and to expand the conditions associated with fewer than 11 deaths in the 2016 analyses. To compare our results with the CDC report, we calculated age-adjusted rates using the direct method and the 2000 US standard population aged 20 to 64 years. All tests of statistical significance were 2-sided with significance set at *P* < .05. Analyses were performed with SAS Enterprise statistical software version 7.12 (SAS Inc). Data analyses were performed from March 15, 2019, through September 23, 2019.

## Results

In 2016, 1 766 790 Medicare enrollees in the 20% sample were aged 21 to 64 years and received their initial Medicare entitlement because of disability. The mean (SD) age of this cohort was 52.2 (10.2) years; 866 914 (49.1%) were women, and 899 876 (50.9%) were men. This 14.9% (95% CI, 14.9%-15.0%) of all enrollees accounted for 80.8% (95% CI, 78.9%-82.7%) of opioid overdose deaths among all Medicare enrollees. The remaining analyses focus on opioid overdose deaths in this Medicare population younger than 65 years who qualified for disability. The opioid overdose mortality rate in this population increased from 57.4 per 100 000 (95% CI, 53.9-61.0 per 100 000) in 2012 to 77.6 per 100 000 (95% CI, 73.5-81.8 per 100 000) in 2016.

Table 1 shows the characteristics and conditions for Medicare enrollees aged 21 to 64 years with a disability entitlement and also for the subset who died because of opioid overdose. In general, those who died from opioid overdose were more likely than the study population to be younger (mean [SD] age, 49.7 [9.9] years vs 52.2 [10.2] years), male (773 [56.4%] vs 899 876 [50.9%]), white (1114 [81.3%] vs 1 170 832 [66.3%]), and eligible for Medicaid (796 [58.1%] vs 866 544 [49.0%]). A larger proportion of these individuals also had psychiatric diseases (depression, 436 [56.8%] vs 264 809 [30.2%]; anxiety, 487 [63.4%] vs 279 297 [30.9%]; and bipolar disorder, 273 [35.6%] vs 122 830 [14.0%]), substance use disorder (tobacco use disorder, 437 [56.9%] vs 207 378 [23.7%]; alcohol use disorder, 186 [24.2%] vs 58 582 [6.7%]; and drug use disorder, 432 [56.3%] vs 105 625 [12.1%]), and chronic pain (489 [63.7%] vs 271 913 [31.1%]). Table 2 presents the results of a stepwise logistic regression model estimating opioid overdose deaths in 2016 among Medicare enrollees younger than 65 years who qualified for disability. In the final models, we found that being male and white, having higher income, coverage with Medicare Part D, enrollment under disability entitlement less than 15 years, and living in metropolitan areas were associated with higher rates of opioid overdose death. Chronic conditions associated with a higher rate of opioid overdose death were associated with substance abuse, including opioid use disorder (adjusted odds ratio [aOR], 3.23; 95% CI, 2.56-4.06), drug use disorder (aOR, 1.86; 95% CI, 1.47-2.37), and tobacco use disorder (aOR, 1.77; 95% CI, 1.49-2.09); psychiatric diseases, including anxiety (aOR, 1.65; 95% CI, 1.37-1.97), bipolar disorder (aOR, 1.51; 95% CI, 1.28-1.79), and depression (aOR, 1.29; 95% CI, 1.09-1.53); and chronic pain (aOR, 1.86; 95% CI 1.57-2.21). Chronic kidney disease (aOR, 1.59; 95% CI, 1.32-1.91), pressure and chronic ulcers (aOR, 1.48; 95% CI, 1.11-1.96), and hepatitis (aOR, 1.68; 95% CI, 1.37-2.05) were also associated with a greater likelihood of opioid overdose death. The C-statistic for the model was 0.863.

**Table 2.** Demographic Characteristics and Medical Conditions Associated With Opioid Overdose Deaths in Medicare Enrollees Aged 21 to 64 Years Qualified for Disability in 2016^a^

Variable	Unadjusted Opioid Overdose Mortality		aOR (95% CI)^b^
	Deaths, No.	Rate Per 100 000 (95% CI)	
Demographic characteristics			
Sex			
Male	413	91.5 (82.9-100.5)	1 [Reference]
Female	353	84.1 (75.6-93.1)	0.76 (0.66-0.89)
Race/ethnicity			
White	637	105.2 (97.2-113.5)	1 [Reference]
Black	57	35.5 (26.9-45.3)	0.45 (0.34-0.59)
Hispanic	39	55.2 (39.2-73.8)	0.61 (0.44-0.85)
Other	33	95.8 (65.9-131.1)	1.07 (0.76-1.53)
Years under disability insurance			
≤4	137	92.6 (77.8-108.8)	1.54 (1.21-1.95)
5-8	317	111.9 (99.9-124.5)	1.64 (1.34-2.00)
9-14	170	95.6 (81.8-110.5)	1.38 (1.10-1.73)
≥15	142	54.2 (45.6-63.5)	1 [Reference]
Zip code income			
Quartile 1 (lowest)	232	76.9 (67.3-87.1)	0.77 (0.62-0.95)
Quartile 2	208	84.7 (73.6-96.6)	0.74 (0.60-0.91)
Quartile 3	167	88.0 (75.2-101.9)	0.72 (0.58-0.90)
Quartile 4 (highest)	159	118.5 (100.8-137.6)	1 [Reference]
Rural vs urban			
Metropolitan	638	97.2 (89.8-104.9)	1 [Reference]
Urban	113	58.9 (48.5-70.2)	0.61 (0.49-0.75)
Rural	15	65.4 (36.6-102.4)	0.68 (0.40-1.15)
Medicare Part D enrollment			
Yes	683	97.8 (90.6-105.2)	1.34 (1.06-1.69)
No	83	48.1 (38.3-59.0)	1 [Reference]
Medical conditions			
Psychiatric diseases			
Depression			
No	331	54.5 (48.8-60.5)	1 [Reference]
Yes	435	165.0 (149.8-180.8)	1.29 (1.09-1.53)
Anxiety			
No	281	46.7 (41.4-52.3)	1 [Reference]
Yes	485	180.3 (164.6-196.7)	1.65 (1.37-1.97)
Bipolar disorder			
No	493	65.8 (60.2-71.8)	1 [Reference]
Yes	273	223.2 (197.5-250.5)	1.51 (1.28-1.79)
Posttraumatic stress disorder			
No	672	81.3 (75.2-87.5)	1 [Reference]
Yes	94	213.0 (172.2-258.2)	0.73 (0.58-0.92)
Substance abuse			
Tobacco use disorder			
No	329	49.5 (44.3-55.0)	1 [Reference]
Yes	437	211.7 (192.3-232.0)	1.77 (1.49-2.09)
Drug use disorder			
No	334	43.6 (39.1-48.4)	1 [Reference]
Yes	432	410.8 (373.0-450.5)	1.86 (1.47-2.37)
Opioid use diagnosis or procedure			
No	416	50.9 (46.1-55.9)	1 [Reference]
Yes	350	645.7 (579.8-715.1)	3.23 (2.56-4.06)
Chronic pain			
Fibromyalgia, chronic pain, and fatigue			
No	278	46.3 (41.0-51.9)	1 [Reference]
Yes	488	180.3 (164.7-196.6)	1.86 (1.57-2.21)
Other diagnoses			
Hypertension			
No	485	96.2 (87.9-105.0)	1 [Reference]
Yes	281	76.5 (67.8-85.7)	0.68 (0.58-0.81)
Hyperlipidemia			
No	630	105.7 (97.6-114.1)	1 [Reference]
Yes	136	49.5 (41.5-58.1)	0.48 (0.39-0.58)
Hepatitis			
No	633	75.6 (69.8-81.6)	1 [Reference]
Yes	133	393.2 (329.2-462.7)	1.68 (1.37-2.05)
Chronic kidney disease			
No	576	80.5 (74.1-87.2)	1 [Reference]
Yes	190	122.2 (105.4-140.2)	1.59 (1.32-1.91)
Chronic obstructive pulmonary disease			
No	633	83.4 (77.0-90.0)	1 [Reference]
Yes	133	118.5 (99.2-139.4)	0.79 (0.64-0.97)
Cancer			
No	750	89.7 (83.4-96.2)	1 [Reference]
Yes	16	46.3 (26.4-71.5)	0.49 (0.29-0.80)
Peripheral vascular disease			
No	722	89.6 (83.2-96.2)	1 [Reference]
Yes	44	67.6 (49.1-89.0)	0.70 (0.50-0.97)
Pressure and chronic ulcers			
No	705	84.8 (78.6-91.1)	1 [Reference]
Yes	61	154.8 (118.4-196.0)	1.48 (1.11-1.96)
Anemia			
No	623	87.1 (80.4-94.1)	1 [Reference]
Yes	143	91.8 (77.3-107.4)	0.79 (0.65-0.97)
Hypothyroidism			
No	709	92.0 (85.3-98.9)	1 [Reference]
Yes	57	56.9 (43.1-72.6)	0.62 (0.47-0.82)
Cataract			
No	750	91.8 (85.4-98.5)	1 [Reference]
Yes	16	29.3 (16.8-45.4)	0.44 (0.27-0.72)
Deafness and hearing impairment			
No	755	89.3 (83.1-95.8)	1 [Reference]
Yes	11	42.8 (21.4-71.5)	0.54 (0.29-0.97)

Abbreviation: aOR, adjusted odds ratio.

^a^ Enrollees with completed data (n = 871 098) were included in the model. There were 4584 (0.5%) enrollees with missing data because of the linkage between their residential zip code and US Census data.

^b^ Odds ratios were calculated from the stepwise logistic regression model, with *P* = .05 to add and remove the predictor. The maximum rescaled *R*^2^ for the model is 0.137. *P*  < .001 from Hosmer and Lemeshow goodness-of-fit test. In general, this test is sensitive to large sample size.

Table 3 shows the opioid overdose mortality rates for the 3 major categories of conditions: substance abuse, psychiatric diseases, and chronic pain. The logistic model including only these conditions with no demographic variables had a C-statistic of 0.82. Among the 37.5% (95% CI, 37.4%-37.7%) of beneficiaries younger than 65 years with Medicare disability entitlement who did not have any of the 3 categories of conditions, the opioid overdose mortality rate in 2016 was 15.5 per 100 000 (95% CI, 11.6-20.1 per 100 000). In contrast, the 11.1% (95% CI, 11.0%-11.2%) of beneficiaries who had all 3 categories of conditions had an opioid overdose mortality rate of 363.7 per 100 000 (95% CI, 326.7-402.6 per 100 000), which is 23.4 times higher than the rate for enrollees without any of the conditions and accounts for 45.8% of all opioid overdose deaths. The 8.1% (95% CI, 8.0%-8.2%) of beneficiaries with substance abuse plus psychiatric diseases without a pain diagnosis had an opioid overdose mortality rate of 187.5 per 100 000 (95% CI, 157.0-220.7 per 100 000), and the 9.3% (95% CI, 9.2%-9.4%) of beneficiaries with pain plus psychiatric diseases without a substance abuse diagnosis had an opioid overdose mortality of 84.6 per 100 000 (95% CI, 65.9-105.7 per 100 000) (Table 3). Overall, 78.0% of all opioid overdose deaths occurred in the 32.0% of enrollees qualified for disability who had at least 2 major condition categories.

**Table 3.** Proportion of Enrollees and the Rate of Opioid Overdose Deaths per 100 000 in 2016 Among Medicare Beneficiaries Aged 21 to 64 Years Who Qualified for Disability With 3 Major Condition Categories: Psychiatric Diseases, Substance Abuse, and Chronic Pain

Major Condition Category			Medicare Enrollees With Disability Entitlement		Opioid Overdose Mortality	
Psychiatric Disease^a^	Substance Abuse^b^	Chronic Pain	No.	Percentage (95% CI)	No.	Rate Per 100 000 (95% CI)
Yes	Yes	Yes	96 790	11.1 (11.0-11.2)	352	363.7 (326.7-402.6)
Yes	Yes	No	70 940	8.1 (8.0-8.2)	133	187.5 (157.0-220.7)
Yes	No	Yes	81 527	9.3 (9.2-9.4)	69	84.6 (65.9-105.7)
No	Yes	Yes	31 383	3.6 (3.5-3.6)	45	143.4 (104.6-188.2)
Yes	No	No	153 257	17.5 (17.4-17.6)	55	35.9 (27.0-46.0)
No	Yes	No	51 000	5.8 (5.8-5.9)	40	78.4 (56.0-104.5)
No	No	Yes	62 213	7.1 (7.0-7.2)	23	37.0 (23.4-53.5)
No	No	No	328 572	37.5 (37.4-37.7)	51	15.5 (11.6-20.1)

^a^ Psychiatric disease included anxiety, depression, or bipolar disorder.

^b^ Substance abuse included tobacco, drug, or opioid use disorder.

To explore the robustness of study results from 2016, we repeated analyses with person-year data from 2012 to 2015. Seven additional conditions (intellectual disability, multiple sclerosis, spina bifida, blindness, traumatic brain injury, acute myocardial infarction, and glaucoma) excluded in the 2016 analysis because of CMS cell size suppression were added in the multivariable analyses because they were associated with 11 or more opioid overdose deaths in the 2012 to 2015 data. The final stepwise logistic regression model from this sensitivity analyses had C-statistics of 0.857 compared with 0.863 in the main analyses. Aside from pressure and chronic ulcers, positive associations with opioid overdose deaths for all other conditions from our main analyses persisted. Three additional conditions—ischemic heart disease, arthritis, and alcohol use disorder—were found to be positively associated with outcomes in the final model of sensitivity analyses.

We compared our results with the CDC report on opioid overdose deaths,^10^ which reported an age-adjusted rate of opioid overdose deaths in the US population in 2016 of 13.3 per 100 000. Assuming that the rate at age 15 to 24 years was the same as that for age 20 to 24 years, the age-adjusted rate was 20.8 per 100 000 for those aged 20 to 64 years in the United States. In contrast, the age-adjusted rate was 99.9 per 100 000 in our study of Medicare enrollees aged 21 to 64 years who qualified for disability, which is at least 4.8 times higher than that of the general US population in 2016.^10^ On the basis of our findings of 1371 opioid overdose deaths in this population from the 20% Medicare sample, we estimated that approximately 16% of the 42 249 opioid overdose deaths in 2016 reported by the CDC occurred in this population.

## Discussion

The finding of increased opioid overdose deaths among adults who qualified for Medicare because of disability is consistent with the CDC report of increased mortality across multiple demographic groups.^10^ The mortality rate in our study is 4.8 times higher than that of the general US population in 2016.^10^ The 37.5% of the study population with no diagnosis of psychiatric diseases, chronic pain, or substance abuse had an age-adjusted rate of opioid overdose death of 15.5 per 100 000, which is lower than that of the US population.^10^ The rate of overdose death greatly increased for patients with any of these 3 conditions, with the highest rate among those with all 3 condition categories.

Previous studies^3,11^ have reported high rates of prescription opioid use among Medicare adults who qualified for disability. In addition to contextual characteristics, such as county income, income disparity, and unemployment rates,^12^ other risk factors associated with higher opioid use in this population were mental illness and chronic pain.^13,14^ The proportion of adult Medicare enrollees with disability entitlement who had a chronic pain diagnosis in our study population (31.1%) was consistent with that of a previous report.^15^ Our finding of an association of Medicare Part D enrollment with opioid overdose death is also consistent with the results from a Veterans Affairs study,^16^ presumably attributable to increased access to prescription opioids.

In the multivariable model, dual-eligible enrollment with Medicaid was not associated with opioid overdose death. We found an unexpected positive association between opioid overdose death and residence in the higher-income zip codes. There is a negative association between income and opioid prescriptions at the county level.^17,18^ At the individual level, a study^19^ based on data from the National Survey on Drug Use and Health also showed that those with lower income were more likely to misuse opioids and had higher rates of opioid use disorder than the general US population. Another study^20^ of the Medicare population showed that enrollees residing in higher-income zip codes had lower rates of long-term opioid prescriptions. It is unclear why higher rates of opioid overdose death were associated with higher income in our study, despite the previously reported lower rate of prescription opioid use in this population. Our findings of higher rates of opioid overdose death in metropolitan areas add additional evidence to the conflict surrounding this topic.^21^ Future studies assessing the association of overdose mortality with income and urban or rural residence stratified by opioid type may give more insight to our unexpected findings.

Subgroups of adults enrolled in Medicare because of disability with different risk profiles for opioid overdose death might benefit from different interventions. For example, the subset of beneficiaries with the lowest rate of opioid overdose deaths likely represents a population for whom prescription opioids can be safely offered by their physicians as part of comprehensive pain management if the clinical condition warrants opioid analgesics. For those at the highest risk of opioid overdose deaths, examples of targeted interventions include case management developed for and tailored to a high-risk population and Medicare-mandated requirement for comanagement by pain, addiction, and mental health specialists in treating pain in these populations. These interventions would have the biggest impact on the subgroups with at least 2 major condition categories that comprised 32.0% of all enrollees who qualified for disability but accounted for 78.0% of all opioid overdose deaths in this population. Patient-informed and evidence-supported comanagement strategies and targeted interventions (including enhanced access to substance use disorder treatment) have the potential to reduce opioid overdose deaths in this high-risk population while ensuring effective pain management.

### Limitations

This study has limitations. First, the quality and accuracy of death certificate data associated with overdose varies across states.^10,22^ In addition, we could not distinguish from our data between deaths deemed accidental, suicide, or homicide or between the deaths that occurred in the inpatient or outpatient setting. Second, we restricted the analyses of associations with medical conditions to enrollees with 2 years of continuous enrollment with fee-for-service coverage. Our results may not be generalizable to health maintenance organization populations. Third, the validity of medical conditions derived from claims data varies. Fourth, we provided descriptive results to show the differences in rates of opioid overdose death across subgroups. We did not conduct analyses to incorporate competing causes of death. Fifth, we did not analyze the association of drug interactions or contaminated street drugs with opioid overdose mortality. Future studies examining the use of pain medications, psychiatric medications, and the 2 in combination is important for understanding why the opioid overdose death rate is much higher in this population. Sixth, we used stepwise regression to find the most important patient characteristics and health conditions associated with opioid overdose mortality in a parsimonious model, with the understanding of the concerns for the bias in parameter and SE estimation.^23^ However, our findings in isolating the 3 major condition categories were robust in our analyses of 2012 to 2015 data.

## Conclusions

Despite the high rates of opioid use in the Medicare population entitled for disability, research^24,25^ shows lower utilization of opioid treatment programs. This population is heterogeneous, with physical and cognitive disorders present at birth along with conditions acquired later in life. The conditions most significantly associated with opioid overdose deaths in our analyses most commonly occur or are recognized in adolescence or later. There is currently a major federal effort to increase access to opioid use disorder treatment programs and to promote knowledge and use of opioid antagonists in cases of suspected overdose. Such programs work best with accurate targeting of populations at high risk. Our findings suggest that straightforward analyses of Medicare data can identify subgroups of Medicare enrollees with the highest rates of death from opioid overdose. Further studies are needed to understand the heterogeneity of medical and psychiatric conditions associated with opioid use, misuse, addiction, and overdose in this population. Such understanding is key to developing specific, data-driven interventions (eg, programs to manage co-occurring substance use disorder and requirement for comanagement by pain, mental health, and addiction specialists) targeted for each subgroup of high-risk populations.
